# Modulation of Zebrafish (*Danio rerio*) Intestinal Mucosal Barrier Function Fed Different Postbiotics and a Probiotic from *Lactobacilli*

**DOI:** 10.3390/microorganisms11122900

**Published:** 2023-11-30

**Authors:** Mark Rawling, Marion Schiavone, Amélie Mugnier, Eric Leclercq, Daniel Merrifield, Andrew Foey, Emmanuelle Apper

**Affiliations:** 1 Aquatic Animal Nutrition and Health Research Group, School of Marine and Biological Sciences, Plymouth University, Plymouth, Devon PL4 8AA, UK; daniel.merrifield@plymouth.ac.uk (D.M.); andrew.foey@plymouth.ac.uk (A.F.); 2 Lallemand SAS, 19 rue des Briquetiers, 31702 Blagnac, France; mschiavone@lallemand.com (M.S.); ameliemugnier@lallemand.com (A.M.); eleclercq@lallemand.com (E.L.)

**Keywords:** postbiotics, lactobacilli, intestinal barrier function, probiotics, zebrafish

## Abstract

It is generally accepted that microbes play a critical role in maintaining gut barrier function, making them ideal to target in order to mitigate the effects of intestinal diseases such as inflammatory bowel disease with specialist supplementations such as probiotic or postbiotic preparations. In this study, specific strains of *Lactobacillus helvictus* both live and inactivated and *Lactobacillus plantarum* inactivated were fed to zebrafish at an inclusion level of 6 × 10^6^ cells/g in order to assess the effects on gut barrier function and protection. Taken together, our results indicate that dietary administration of pro- or postbiotics strengthens the gut barrier function and innate immunity of healthy zebrafish in a strain-specific and process-dependent way. With some differences in the response intensity, the three treatments led to increased intestinal villi length and proportion of IELs, reinforcement of the GC population and up-regulated expression of biomarkers of AMP production and tight junction zona-occludin 2a (*zo-2a*). In addition, LPPost had an impact on the adaptive immune response, and we hypothesized that it conferred the potential to drive Th17/ILC3 immunity, as suggested by its effect on the gene expression of *il22*, of different AMPs, and the expression of *zo2a*. Moreover, LPPost showed the potential to drive Th1/ILC1-like immunity, with a higher percentage of CD8^+^ cells and higher *ifnγ* gene expression. In summary, the use of inactivated *Lactobacilli* species in this study represented a promising strategy for improving barrier function and regulating the immune fate of the intestinal mucosa in a strain-specific way.

## 1. Introduction

The intestinal mucosa is a highly specialized mucosa that allows for the digestion and absorption of nutrients while maintaining the coexistence between the host and microbiota, allowing for enhanced protection against infection [1]. Thus, the gut barrier function, referring to the chemical and physical components required to fulfill this function, plays a crucial role not only in intestinal homeostasis but also in governing systemic health, illustrating the famous sentence of Hippocrates, “*all disease begins in the gut*”. In that sense, several reviews have reported an association between the impairment of the gut barrier function parameters and several diseases, among which are inflammatory bowel disease (IBD) through pro-inflammatory cytokine-mediated responses [2], obesity and/or type 2 diabetes through innate chemokine signaling, eosinophils, immunoglobulin A (IgA), T helper (Th) 17 cells and their cytokines [3], food allergy diseases [4], and even skin diseases [5]. Besides this, the proper functioning of the gut barrier is important to the bidirectional signaling between the enteric nervous system and the brain, i.e., the “*gut–brain*” axis. Thus, because of its biological importance, it appears crucial to develop new nutritional strategies to reinforce gut barrier integrity and to prevent or mitigate its potential dysregulation. Although gut barrier dysfunction can be related to certain abiotic factors such as diet, host genetics, or other environmental factors, it is accepted that microbes play a critical role in maintaining gut barrier function, making them ideal to target with specialist supplementations such as probiotic or postbiotics preparations.

In this context and due to their modes of actions, pro- and postbiotic bacteria appear to be good candidates to sustain gut barrier function. A probiotic is defined as live microorganisms which when administered in adequate amounts confer a health benefit on the host [6], while a postbiotic is defined as a preparation of inanimate microorganisms and/or their components that confers a health benefit on the host [7]. However, for the later, no true consensus has been accepted worldwide, and alternative words such as paraprobiotics, ghost probiotics or tyndallised bacteria can be found in the current literature. Bacteria, among which are recognized probiotics like *Lactobacillus, Bifidobacterium* or some strains of *Escherichia coli*, play a key role in influencing gut barrier function [8]. Indeed, they possess multiple types of microbial-associated molecular patterns (MAMPs) such as flagella, pilli, surface layer proteins, lipopolysaccharide, peptidoglycan, and teichoic and lipoteichoic acid that interact with innate pattern recognition receptors (PRRs) like toll-like receptors (TLRs) and NOD-like receptors [9]. Probiotics can also produce different metabolites such as secreted proteins, organic acids, indoles, and bacteriocins that can boost the production of mucus by modulating goblet cell (GC) dynamics or influence gut mucosa metabolism [10]. Interestingly, both live and inactivated bacteria, in particular *L. helviticus* and *L. plantarum*, show differential effects on TLR signaling and tight junction protein regulation to maintain gut homeostasis in humans and mice [11,12,13,14,15]. While inactivated forms (postbiotics) can be easier to use for certain applications in the food and feed industry by providing the advantages of longer product shelf life or stability, easier storage, and better stability during harsh feed production regimes, the effects are strain specific. Studies have shown that different signaling pathways such as nuclear factor kappa B (NF-kB), AP-1 transcription factor or mitogen-activated protein kinases (MAPKs) can be activated depending on the strain [16,17]. In addition, their effects can differ depending on whether bacteria are alive or inactivated [18]. *L. helviticus* and *L. plantarum* are two well-known probiotic species used in human food medicine; however, their use in animal feed production is not so well known. Therefore, characterization of the effects of each strain of pro- or postbiotics on gut barrier function is thus required to allow for specialist strategic implementation and to improve the concepts of personalized nutrition and immunonutrition.

An accurate and validated integrative model is consequently required to characterize the gut barrier function and to objectivize the strain-specific mechanisms involved with live or inactivated bacteria. Among the different available integrative models, the zebrafish is an attractive organism that is readily utilized to perform translational research on metabolic diseases, digestive health, immunity, and cancer. Its small size and high fecundity rates as well as the full annotation of its genome are undoubtedly advantageous. In addition, zebrafish is well-recognized to specifically study the gut barrier function for several reasons, among which are the large conservation of PRRs reacting to microbes in zebrafish and mammals [19], the numerous conserved pathways in immunity, gut epithelial homeostasis and inflammation [20], the presence of innate and adaptive immunity, and the fact that it is possible to study underlying pathways of human/animal diseases like type 2 diabetes or IBD. Hence, this organism can be used as a good comparative species to elucidate the modes of action involved in biotics supplementation.

The aim of this study was to substantiate the effects of a strain of live *L. helveticus* (strain: HA-122), and to compare the effects of two different heat-inactivated bacteria, *L. helveticus* HA-122 and *L. plantarum* HA-119, on the gut barrier function of healthy adult zebrafish that were not subjected to challenge. Our hypothesis was that the pro- and postbiotics would be able to strengthen the gut barrier function through crosstalk with the intestinal epithelial cells and that this crosstalk differed depending not only on the bacterial strain used, but also on the process applied to manufacture such strains (e.g., live or inactivated forms). With this information, it could be possible to select the best candidate (s) to be used in case of challenges.

## 2. Materials and Methods

### 2.1. Test Products and Experimental Diets

The test products consisted of proprietary, commercially available, single-strain lactic acid bacteria provided by Lallemand Health Solution (LHS, Blagnac, France), namely *Lactobacillus helveticus* HA-122 and *Lactobacillus plantarum* HA-119. The former was provided and tested in live and heat-inactivated form (LHPro and LHPost, respectively); the latter was provided and tested heat-inactivated only (LPPost). Probiotics were produced and heat-inactivated according to internal proprietary protocols. LHPro and LHPost originated from the same production batch with LHPro being provided in the lyophylised form.

A basal diet was formulated (Table 1) using feed formulation software (Feedsoft^®^, version 10.1) in order to meet the known nutritional requirements of cyprinids [21]. The basal diet was produced by mechanically stirring the ingredients into a homogenous mixture using a Hobart food mixer (Hobart Food Equipment, Hobart, Australia, model no: HL1400—10STDA mixer). Warm water was added to reach a consistency suitable for cold press to form pellets (PTM Extruder system, model P6, Termoli, Italy). The experimental diets were produced by spraying with *Lactobacillus helveticus* HA-122, either in a live (LHPro) or heat-inactivated (LHPost) form, or with heat-inactivated *Lactobacillus plantarum* HA-119 (LPPost) in sterile PBS (pH 7.0) onto the cold press-extruded basal diet and allowing to dry for 24 h at room temperature. 

Each test ingredient was incorporated at a concentration of 6 × 10^6^ cells/g of feed. Diets were then dried, ground, and sieved to isolate pellets of ϕ600–800 µm for the trial. The nutritional profile of the diets was determined according to AOAC protocols [22] (Table 2).

### 2.2. Characterization of Lactobacilli

To image the topography of each heat-inactivated Lactobacilli, atomic force microscopy (AFM) images were recorded on an AFM Nanowizard III (Bruker, Billerica, MA, USA) in contact mode in PBS at room temperature with a force of 1 nN. MLCT cantilevers (Bruker, Billerica, MA, USA, nominal spring constant of ~0.01 N/m) were used with a scanning rate of 1 Hz and a resolution of 128  ×  128 pixels. The images acquired were analyzed using the Data Processing software from JPK Instruments (Bruker, Billerica, MA, USA). Lactobacilli were fixed with 2.5% glutaraldehyde in PBS, dehydrated stepwise with increasing concentrations of ethanol 30–100%, and then imaged by scanning electronic microscopy with a Quanta 250 FEG (FEI, Hillsboro, OR, USA). 

The zeta-Potential measurements of heat-inactivated cells and the size measurement of microvesicles (MVs) were performed using a ZetaSizer NanoZS (Malvern Instruments Ltd., Worcestershire, UK). Three runs of the measurements at 25 °C were performed for each sample to obtain the zeta potential. Cells were resuspended in 1 mM KNO_3_ at concentrations of about 2 × 10^8^ CFU/mL. The pH was adjusted with KOH or HNO_3_, with a new suspension made for each determination. After the rehydration for 1 h at 4 °C of 1.2 × 10^7^ CFU/mL heat-inactivated *Lactobacillus*, MVs were isolated as described elsewhere [23]. For size measurements, MVs were used at a 10-fold dilution from stock concentration. Proteins were separated on a SDS-PAGE gel (12%) following Coomassie Blue staining.

### 2.3. Experimental Design and Feeding

The experiment was carried out at the Aquaculture and Fish Nutrition Research Aquarium at the University of Plymouth (Plymouth, UK) and was approved by the University of Plymouth’s animal ethical review board under number ETHICS-32-2019. A 5-week trial was conducted during which zebrafish were fed one of four diets in triplicate tanks: (1) Control (non-supplemented basal diet), (2) LHPro, (3) LHPost, or (4) LPPost. The system was an indoor freshwater recirculated aquaculture system (RAS) equipped with mechanical, biological filtration, temperature control and aeration. The RAS system consisted of 15 rectangular tanks (15-L tanks) each with a water flow rate set at 15 L/h. Wild-type (WIK) zebrafish stocks were originally sourced from the European zebrafish resource centre (EZRC, Eggenstein-Leopoldshafen, Germany); F2 generations were bred from the original stock and used in the experiments. Sentinel fish were routinely screened for pathogens using molecular diagnostic analysis to ensure they were disease free (Surrey Diagnostics, Cranleigh, UK). For this trial, 300 male zebrafish were randomly distributed into the experimental system (25 fish/tank, initial mean bodyweight (BWi) = 0.50 ± 0.06 g) at the beginning of the trial. During the trial, fish were kept under the same experimental conditions as published in Rawling et al. [24]. 

Fish were hand-fed at 4.0% biomass per day, and husbandry and feeding regimes followed the same protocols as outlined in Rawling et al. [24]. 

### 2.4. Tissue Preparation and Light Microscopy

At the end of the trial, 3 fish per tank (9 fish/treatment) were dissected for histology as follows. Posterior intestinal (PI; defined in Supplementary Figure S1) samples were excised and digesta was removed using the same protocols and reagents as outlined in Rawling et al. [24]. 

For light microscopy, formalin-fixed PI samples were prepared according to the methods outlined in Rawling et al. [24]. Multiple consecutive sections at 3 µm in thickness for each sample were stained with haematoxylin and eosin (H&E) to assess intestinal morphometry after Rawling et al. [24], and Alcian blue-period acid Schiff (AB-PAS) stain to assess goblet cell density (GCD), goblet cell coverage (GCC) and goblet cell mucin chemotype. GCD was calculated by counting the number of goblet cells per 200 µm of villi tissue. GCC was calculated from the cumulative area of total goblet cells counted for each villus/total area of villi multiplied by 100 as in Leclercq et al. [25]. Intraepithelial leukocyte (IEL) density was calculated by counting the number of IELs per 100 enterocyte cells. Quantitative measurements of each image were taken using Image ‘J’ 1.47v software (National Institutes of Health, Bethesda, Rockville, MD, USA). 

### 2.5. Intestinal Tissue Lysozyme and Cathepsin L

The PI intestines of 2 fish per tank (6 fish/treatment) were dissected and analysed by indirect ELISA for the detection of Lysozyme and Cathepsin L [26]. Briefly, the PI were homogenized on ice in Tris-Triton lysis buffer (10 mM Tris, 100 mM NaCl, 1 mM EDTA, 1% *v*/*v* Triton X-100, and protease inhibitor cocktail x1, pH = 7.4). Subsequently, the homogenate was centrifuged at 12,000× *g* for 20 min at 4 °C. The supernatant containing the soluble proteins was stored at −20 °C until further analysis. The protein concentration of each sample was quantified by using the BCA protein assay kit (Thermofisher Scientific, Paisley, UK) following the manufacturers’ instructions. Samples were then diluted in carbonate bicarbonate buffer (0.2% *w*/*v* Na_2_CO_3_ and 0.3% *w*/*v* NaHCO_3_, pH 9.6) and seeded in duplicate in a 96-well plate (Maxisorp, Nunc, Thermofisher Scientific, Paisley, UK) at 20 µg/mL (100 µL/well) for overnight incubation at 4 °C. Samples were then blocked with 1% Bovine serum albumin for 2 h at room temperature (250 µL/well). For the capture of lysozyme, the primary antibody was added at a dilution of 1:1000 (Anti-lysozyme polyclonal antibody, Abcam, Cambridge, UK) and incubated at room temperature for 60 min (100 µL/well). For the capture of cathepsin L, the primary antibody was added at a dilution of 1:1000 (Anti-Cathepsin L/MEP polyclonal antibody, Abcam, Cambridge, UK) and incubated at room temperature for 60 min (100 µL/well). After washing, the secondary antibody was added at a dilution of 1:1000 (Goat Anti-Rabbit IgG conjugated with HRP, Abcam, UK) and incubated at room temperature for 60 min. Finally, after washing, the chromogenic substrate was added, 3,3′,5,5′-tetramethylbenzedine (Sigma Aldrich, Merck, Gillingham, UK), and incubated at room temperature for 10 min (100 µL/well). The reaction was stopped with 50 µL of 1 N sulfuric acid, and absorbance at 450 nm was measured using a Spectramax microplate reader (Molecular devices, San Jose, CA, USA). 

### 2.6. Gene Expression Analysis

PI samples from 6 fish per tank were pooled into two samples (*n* = 6/treatment), and RNA extraction and cDNA synthesis were performed according to previously published protocols [24]. 

The real-time PCR assay was performed according to previous protocols [24]. The primers used and their sequences are presented in Table 3. *Cops2* and *metap1* were used as reference genes in each sample in order to standardize the results by eliminating variation in mRNA and cDNA quantity and quality [27]. The stability of *cops2* and *metap1* was determined using the same protocol as outlined in Rawling et al. [24]. PCR efficiencies for primer sets were determined and presented using the equation E (PCR efficiency) = 10(−1/slope; Table 3). The expression of target genes was displayed as fold change (FC (Log2).

### 2.7. Flow Cytometry of Intestinal Lymphoid Cells

Dissected PI from 8 pooled fish per sample and 2 samples per tank were first stored in L-15 medium (Life Technologies, Paisley, UK) with 2% Fetal Bovine Serum (FBS; Sigma-Aldrich, Gillingham, UK), and 100 U/mL penicillin and streptomycin (Thermofisher Scientific, Paisley, UK) at 4 °C until further determination of helper and cytotoxic (CD4^+^ and CD8^+^, respectively) T-cells proportions. Samples were then immediately treated for 1 h at 37 °C with Liberase enzyme mixture to facilitate the dissociation of cells (Roche, 0.2 U/mL in phosphate buffer saline, pH 7.0). Further dissociation of cells from tissue was performed manually by passing samples through a 70 and 40 µm cell strainer (Pluriselect, Cambridge, UK). Cells were then centrifuged at 400× *g* for 5 min at 4 °C and resuspended in L-15 media with 2% FBS and kept on ice until analysis (1 × 10^6^ cells/sample). Cells were washed in Trizma buffer saline (TBS; pH 7.0) and were single stained with CD8 Alpha Chain mab [28] (CD8α, clone 2C3, Cosmo Bio, Carlsbad, CA, USA) and for surface glycoprotein CD4 mab [29] (clone 6D1, Cosmo Bio, Carlsbad, CA, USA) primary antibody diluted 1:500. After washing with TBS, cells were stained with corresponding secondary antibodies: Alexa Flour-488 conjugated donkey-anti-rat (CD8α) or Alexa Flour-647 conjugated donkey-anti-rat (CD4), secondary antibodies were diluted 1:1000 (Abcam, Cambridge, UK). Flow cytometry was performed using a FACSAria II Flow cytometer (BD Biosciences) and was analysed using the FlowJo v10 software (BD Bioscience, Berkshire, UK). Dead cells were excluded from the analysis using propidium iodide viability stain at a final concentration of 2 µg/mL (Sigma-Aldrich, Gillingham, UK). Forward and side scatter profiles were used to gate on the lymphocyte population, and the gating strategy is shown in Supplementary Figure S2. Compensation controls for each fluorochrome conjugated secondary antibody were performed using the AbC™ total antibody compensation bead kit according to the manufacturer’s instructions (Life Technologies, Paisley, UK). 

### 2.8. Statistical Analysis

All statistical analyses were carried out using R version 3.4.1 [30]. Rt-qPCR data were analysed using the non-parametric 2-sample permutation test [31]. Gene expression data showing comparisons with the control group are presented as means ± SEM (fold change (Log2)). Histological parameters for GCC, GCD, Acid mucins, neutral mucins, both mucins and intraepithelial lymphocytes (IELs) were analysed using the non-parametric 2-sample permutation test. All other data were assessed by one-way ANOVA tests with Tukey HSD post-hoc analysis where differences occurred, data are presented as mean ± standard error of the mean (SEM). The significative differences between control and experimental groups were accepted at *p* < 0.05, a trend was considered when *p* < 0.10.

Two principal component analyses (PCA) were performed to summarize the information contained in the selected sets for mucosal barrier protection biomarkers and immunity biomarkers and to evaluate the pattern of their relationships [32]. All PCA were conducted using FactoMineR with a shiny application [33]. The relevant dimensions of each PCA were selected using scree plots, which are based on eigenvalues.

## 3. Results

### 3.1. Characterization of Postbiotics

**Figure 1:**
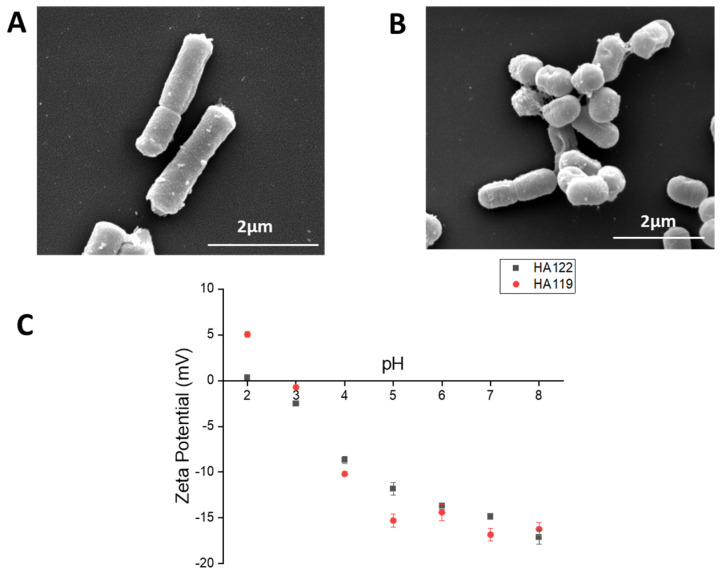
Morphology and surface charge of LHPost and LPPost. (**A**,**B**) Scanning electronic images of two cells of LHPro and LHPost revealing extracellular membrane vesicles at the proximal of the cell wall (**C**) Zeta-potential measurements (mV) of LHPost and LPPost at different pH.

The cell morphology of heat-inactivated *L. helveticus* and *L. plantarum* was examined by electronic microscopy. As can be observed (Figure 1A), both cells displayed a rod cell shape, revealing that the cell shape of each lactic acid bacteria remained intact even after heat inactivation. Moreover, it was observed that the cell wall integrity of each lactic acid bacteria was unaffected. 

The measurement of the zeta-potential as a function of pH of each heat-inactivated bacteria was assessed in order to determine the values of isoelectric point (pI) (Figure 1B). *L. helveticus* containing an S-layer in its cell wall [34] has a pI of 2.2, while *L. plantarum* has a pI of 3.7. The interpretation of the zeta-potential in terms of surface charge densities is difficult for microorganisms, but it can be assumed that bacterial outer cell wall components mainly contribute to the surface charge, which can lead to a qualitative description of the main outer component present on the bacteria [35]. Gram-positive cell wall lactic acid bacteria are composed of an inner layer of peptidoglycan and an outer layer containing different structures: lipotechoic acids, polysaccharides that can be neutral or acidic, and surface proteins such as highly basic S-layer proteins (pI > 9). 

The low pI values for heat-inactivated *L. helveticus* and *L. plantarum* and the observation that the zeta-potential is negative for the entire pH range (pH 3–8) indicate that anionic polysaccharides are the main constituents of the outer cell wall of both lactic acid bacteria. Interestingly, on the cell surface of *L. plantarum*, some vesicles were observed by electronic microscopy (Figure 1) as well as by atomic force microscopy (Supplementary Figure S3). To determine whether MVs were present on heat-inactivated bacteria, inactivated *L. plantarum* and *L. helveticus* were rehydrated, and cell-free supernatant was used further for the isolation of MVs. Analysis of the vesicles using dynamic light scattering revealed that these vesicles have a size of 38–91 nm (median of 77 nm) for *L. helveticus* and 91–712 nm (mediane of 160 nm) for *L. plantarum*, which is similar to the size of outer membrane vesicles (MVs) already observed in live bacteria [36] (Supplementary Figure S3). SDS-PAGE gels of these vesicles show some slight bands at 75 kDa and 37 kDa, which are similar to the bands observed for purified MVs from *L. casei* [37].

### 3.2. Morphometric and Goblet Cell Chemotyping Show Strengthening of Mucosal Barrier

Light microscopy of the posterior intestine revealed healthy tissue (Supplementary Figure S4), and muscularis thickness was consistent across groups. Lamina propria width was significantly lower in the LHPost compared to all other groups, while villi length was significantly higher in the LHPro and LPPost groups compared to the control (Table 4).

There were significant diet effects on the goblet cell dynamics. The LPPost group was characterized by significant elevations of GCD and GCC and by the proportion of acidomucins compared to the LHPro and control groups (Table 4). The LHPost group showed a significantly higher GCC compared to the LHPro group, and a higher proportion of acidomucins compared to the control group (Table 4). Compared to the control regime, a significantly lower GCC and no change in GCD were observed in fish fed the LHPro diet (Table 4). In accordance with these findings, there was a trend towards elevated expression of *muc2.1*, a major gel-forming mucin of the colon forming a protective gel barrier, in LHPro and LPPost compared to the control and LHPost groups (Supplementary Figure S5). 

IEL abundance was significantly higher in all diets compared to the control (LHPro: +30.1%; LHPost: +76.1%; LPPost: +29.6%, *p* < 0.001 for all comparisons) and was also significantly higher in LHPost compared to both the LHPro and LPPost diets (+35.3%, *p* = 0.002 and +35.9%, *p* = 0.001, respectively). 

**Figure 2:**
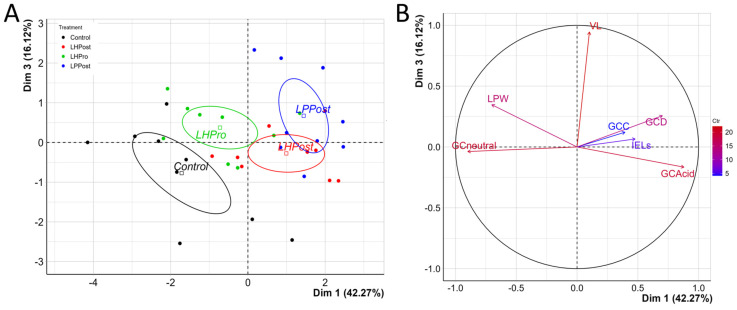
Elevated phenotypic markers showing the fortification of the mucosal barrier in fish fed diets supplemented with postbiotics compared to other treatments. (**A**) PCA plot of individuals and their distribution that was significantly affected by the mucosal barrier protection variables as indicated in plot B. Confidence ellipses were drawn around the levels of the categorical variables for treatment status with a confidence level of 0.95. (**B**) Variables plot showing the highest (red) to lowest (blue) contribution of mucosal barrier protection variables affecting the distribution of individuals, as shown in PCA plot A. Specific terms on plot B are: GCneutral = neutral mucin goblet cells; GCAcid = acidic mucin goblet cells; GCC = goblet cell coverage; GCD = goblet cell density; IELs = intraepithelial leukocytes, LPW = lamina propria width, and VL = villi length.

Two main axes were used for the PCA analysis of all of the data associated with histological parameters, with a total inertia of 76.0% (42.3% for the Dim1 and 16.1% for the Dim3; Figure 2A). Variables related to acidomucin chemotype, GCD, IELs and GCC contributed to the first axis (Dim1), all being positively and significantly correlated with Dim1 (Figure 2B and Figure S5). Variables related to VL and LPW contributed to the second axis (Dim3), both being positively and significantly correlated with Dim3 (Figure 2B and Figure S5). The PCA analysis revealed that each experimental group had its own pattern, with four distinct ellipses and a significant treatment effect according to the permutation-based statistical test (Figure 2A, *p* < 0.001). While the control group was characterized by higher neutral mucin chemotypes with negative coordinates on the first axis, the LHPost group was characterized by higher acidomucin chemotype, GCC, and IELs. Moreover, LPPost was mainly correlated with a high percentage of acidomucin chemotype and GCD (Figure 2A,B).

**Figure 3:**
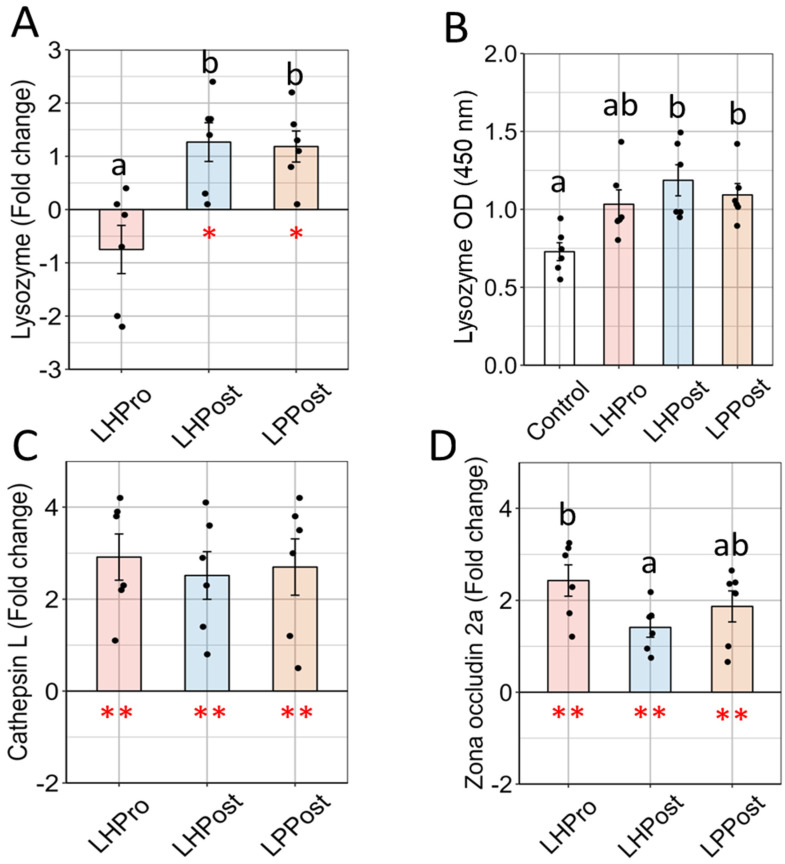
Gene expression analysis revealed positive elevations in markers, suggesting a fortification of the mucosal barrier in fish fed diets supplemented with postbiotics compared to other groups. (**A**) Lysozyme gene expression (fold change relative to the control (Log2)); (**B**) Lysozyme; (**C**) Cathepsin L and (**D**) Tight junction protein zona occluding 2a gene expression. Data are presented as means ± SEM. Asterisks denote significance in gene transcription compared to the control diet (* *p* < 0.05; ** *p* < 0.01). Different letters denote significant differences in gene transcription among the three diets (LHPro, LHPost, and LPPost) for each target gene (*p* < 0.05).

Lysozyme gene expression was significantly up-regulated compared to the control in the LHPost (+63.9%, *p* = 0.02) and LPPost groups (60.4%, *p* = 0.02) (Figure 3A). Likewise, there was a significant increase in lysozyme protein level (indirect ELISA) in both the LHPost (+38.6%, *p* = 0.004) and the LPPost groups (+33.3%, *p* = 0.02), and a trend (*p* = 0.07) in the LHPro group compared to the control (Figure 3B). 

The gene expressions of both Cathepsin L and Zona Occludin 2a were significantly upregulated in all groups compared to the control (Figure 3C,D). The gene expression of *cathL* was similar between the LHPro, LHPost and LPPost groups, while *zo2a* was significantly higher in the LHPro group than in the LHPost group (*p* = 0.04). 

### 3.3. Mobilization CD4/CD8 Positive Cells

**Figure 4:**
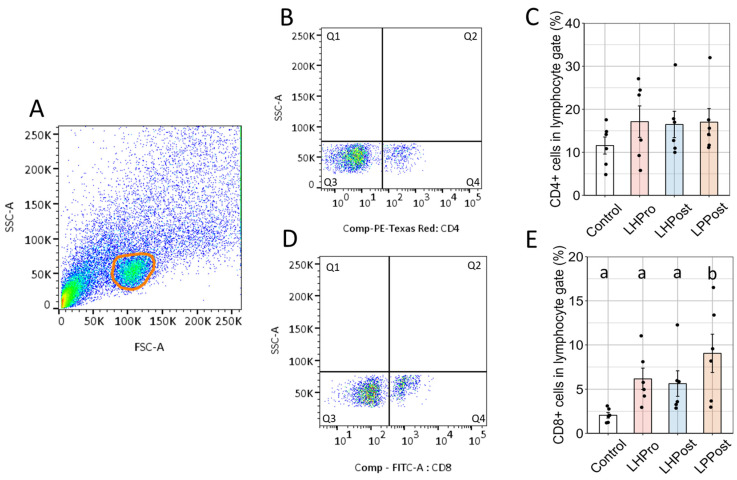
Mobilization of positive CD8α^+^ cells in the posterior intestine of zebrafish fed probiotic and postbiotic treatments. (**A**) Lymphocyte gate; (**B**) positive CD4 cells as indicated by population in Q4; (**C**) prevalence of positive CD4 cells in the lymphocyte gate; (**D**) positive CD8 cells as indicated by population in Q4; (**E**) prevalence of positive CD8 cells in the lymphocyte gate. Data are presented as the mean ± SEM; different letters indicate significant difference between experimental groups (Tuckey post-hoc test; *p* < 0.05).

In order to assess whether live or heat-inactivated bacteria can modulate the proliferation of CD4^+^ and CD8^+^ T cells in the posterior intestine of zebrafish, the lymphocyte gate was analysed using flow cytometry (Figure 4A). The proportion of CD8α^+^ T cells was significantly higher in the LPPost group compared to all other treatments (*p* < 0.01).

### 3.4. Modulation of Innate Intestinal Immune Responses by Postbiotic Groups

**Figure 5:**
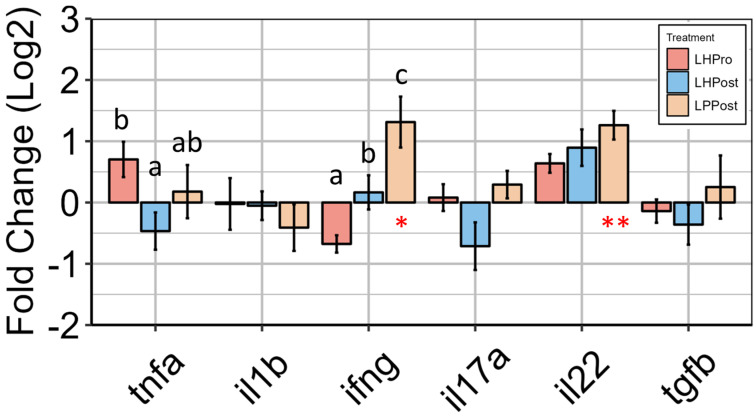
Gene expression analysis reveals the modulation of innate immune effector cytokines by postbiotic groups in the posterior intestine of zebrafish. Relative expression level (fold change (Log2)) to the control group of innate immune markers is shown. Data are presented as mean ± SEM. Symbols in red denote significance in gene expression compared to the control group (* *p* < 0.05; ** *p* < 0.01). Letters denote significant differences in gene expression among the experimental groups (LHPro, LHPost, and LPPost) for each target gene (*p* < 0.05).

The gene expression level of several innate immune markers, including *il4*, *tnfα*, *il1β*, *il17a*, *ifnγ* and *il22*, was analyzed. There was a significant up-regulation in the expression of *ifnγ* in the LPPost group compared to all the other groups (Figure 5). The gene expression of *il22* (+65.0%, *p* = 0.01) was also found to be upregulated in the LPPost group compared to the control group (Figure 5D).

The expression of *tnfα* was not different between treatments and the control group but was significantly higher in the LHPro compared to the LHPost group (Figure 5). The gene expression of *il17a* and of *il1β* was unaffected by the different treatments (Figure 5).

### 3.5. PCA Analysis of Immunity

Two main axes were used to analyze innate immune markers, antimicrobial protein and tight junction data, with a total inertia of 47.8% (32.2% for Dim1, and 15.6% for Dim2. Variables related to the gene expression of *il22*, *tgfβ*, *muc2.1*, and *cathl* strongly contributed to the first axis, all being positively and significantly correlated with Dim1 (Supplementary Figure S5). The variables CD8 and cathepsin L contributed significantly to the second axis, both CD8 and cathepsin L being positively correlated; while the expression data for *muc2.1* and *tnfα* negatively correlated to this axis (Supplementary Figure S5). 

**Figure 6:**
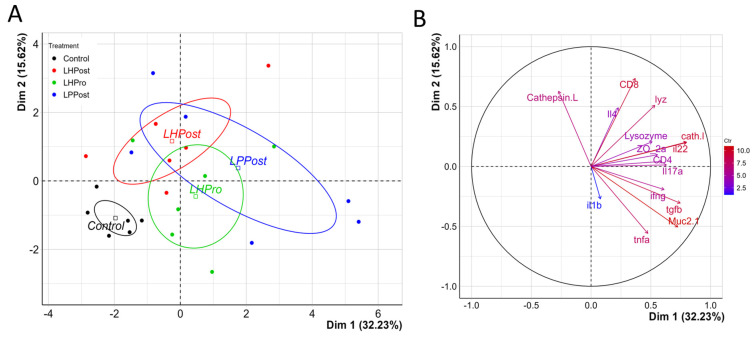
Positive differentiation of immune response markers as shown by principal component analysis. (**A**) PCA plot of individuals and their distribution that was significantly affected by the immune response variables as indicated in (**B**). Confidence ellipses drawn around the levels of the categorical variables for treatment status with a confidence level of 0.95; variables plot showing the highest (red) to lowest (blue) contribution of variables to the PCA plot (**A**). Cathepsin L and Lysozyme indicate markers measured by ELISA; immune gene expression markers are indicated in lower case; CD8 and CD4 indicated specific T-cell markers for adaptive immune cell phenotypes (CD8α^+^ and CD4^+^ cells, respectively); tight junction and mucus biomarkers were indicated by ZO- 2a and Muc2.1, respectively.

The PCA analysis revealed that each group of treatments had its own pattern, with three overlapping ellipses and one distinct ellipse and a significant treatment effect between the control and experimental groups according to the permutation statistical test (Figure 6B, *p* = 0.002), LPPost being the most different group when compared to the control, followed by LHPost and LHPro. While the control group was characterized by a strong negative correlation to all variables on the first axis, the LPPost group was characterized by strong positive correlations to variables CD8, *cath l*, *il22* and *muc2.1* (Supplementary Figure S5).

## 4. Discussion

*Lactobacillus*, live or inactivated, has been widely used in clinical studies and in dairy foods for the prevention and treatment of various gastrointestinal infections and inflammatory conditions. It is thus of importance to provide scientific evidence of the efficacy of pro- and postbiotics at a strain-level and to verify whether the industrial heat treatment applied still confers host benefits. We documented the use of inactivated Lactobacilli species in this study, conferring a promising strategy for improving barrier function and regulating the immune fate of the intestinal mucosa in a strain-specific way. 

Intestinal morphological analysis revealed that all the fish were healthy. Experimental groups LHPro and LPPost resulted in higher villi length compared to the control regime, which could be due to a trophic effect on epithelial cells and lead to a potentially greater surface area for absorption [38]. It is supposed that probiotics activate mitotic cell division and induce the proliferation of gut epithelial cells, which may explain the increase in villus length [39]. Indeed, inactivated *Lactobacillus sakei* HS-1 fed to broiler chickens resulted in hypertrophic intestinal absorptive epithelial cells [40]. This suggests that both live and inactivated bacteria can be effective to create a trophic effect on villi and lead to a change in the gut ultrastructure and a greater surface of absorption, maybe by acting on mitotic and apoptotic signalling pathways, even if the exact mechanisms remain to be elucidated. Some authors reported that IL-22 drives epithelial cell proliferation [41], which would agree with our findings. 

Gut permeability refers to the rate of flux of molecules across the epithelium and its increase, also called “*leaky gut*”, and is associated with many gut disorders. Addition of pro- and postbiotics resulted in higher gene expression of *zo2a*, an intracellular scaffold protein involved in the maintenance of gut permeability. Indeed, Ni et al. [42] reported that dietary supplementation of zebrafish subjected to TNBS-induced colitis with *Lactobacillus casei* 122 resulted in upregulation of *claudin 11* gene expression. In humans, live *L. plantarum* is recognized by TLR-2 which, in turn, may activate protein kinase C (PKCα and γ) and leads to an increase in transepithelial resistance and a redistribution of *zo-1*. Jariwala et al. [43] reported that live *L. helveticus* FA-7 was able to significantly increase the mRNA expression of *zo1* and *clau1* and to improve protein distribution after an EPEC O26: H11-induced epithelial barrier dysfunction in vitro using Caco-2 and HT29 cells. These studies confirm that the effects of *Lactobacillus* are strain-specific and probably involve different mechanisms, through direct cell-to-cell interaction, so a precise characterization of bacterial cell walls is warranted to understand the modes of action. 

In addition to tight junctions, modulation of mucus production by GCs is an important factor in preserving gut epithelial barrier integrity. Intestinal microbes may affect GC dynamics and the mucus layer directly via the local release of bioactive factors or indirectly via the activation of host immune cells. In the current study, GCD and GCC increased with LHPost and LPPost, and this was associated with a significantly higher proportion of acidomucin-producing GC. The use of the two postbiotics thus resulted in a rearrangement of the GC functions towards production of acidic mucins to protect against bacterial translocation via fortification of the mucus barrier by modifying mucus viscosity and buffering capacity [44]. Several studies reported a positive effect of different strains of lactobacilli, either live or inactivated, on the number and the chemotype of GC in various animal species, including zebrafish, subjected to intestinal challenges [45,46,47]. Interestingly, exopolysaccharides extracted from a strain of *L. plantarum* NCU116 have been shown to enhance GC differentiation and to promote the expression of the *muc*2 gene of colonic cells of a mouse subjected to a colitis challenge [48], emphasizing that bacterial surface molecules are involved in this effect. In addition, numerous mechanisms for adhesion to mucus have been discovered in lactobacilli, including mucus binding proteins. In the study of Ni et al. [42] using a TNBS-induced colitis zebrafish model, *L. casei* 122 supplementation resulted in an up-regulation of *muc2* gene expression. In the current study, *muc2.1* gene expression was elevated in both LHPro and LPPost, although not significantly. In contrast to the literature, our study utilized healthy animals, so there was no mucus damage. This study highlights the potential of postbiotics to modulate GC differentiation and the need to finely characterize the regulatory networks that interface with GC dynamics to utilize pro- and postbiotics, as mucus alterations appear to characterize most diseases of mucosal tissues.

Antimicrobial peptides (AMPs) are an integral part of the innate immune response in all multicellular organisms and represent a key aspect of barrier protection [49]. Besides their direct bactericidal activity, they constitute a link between innate and adaptative immunity and are critical to maintaining gut homeostasis. Our findings show positive elevations in lysozyme gene and protein expression alongside elevations in the expression of *cathepsin L* and *zona occludin 2A* in the postbiotic groups compared to other experimental treatments. Cathepsin, among other factors, is involved in the regulation of tight junction proteins such as occludin and claudins. Moreover, lysozyme is highly conserved in the animal kingdom and plays an important role in the protection of the host from potential bacterial and viral infection. Today, there are only a limited number of studies that compare the capacity of candidate probiotic/postbiotic strains to induce such peptides’ expression, and much of the work has been conducted in vitro, notably using the Caco-2 epithelial cell line [50,51,52,53]. Despite this, in healthy adult male zebrafish, *lyz* together with *tnfα* gene expression increased in groups fed with live *Lactobacillus casei* probiotic compared to the control, interestingly in a dose-response manner from 10^5^ to 10^7^ CFU/g feed. 

It is recognized that probiotics from the genera *Lactobacillus* modulate cytokine gene regulation in a strain-specific way. Indeed, germ-free larvae of zebrafish immersed with *Lactobacillus fermentum* NA4 displayed an increased *il10* gene expression and a decreased *il1β* and *tnfα* gene expression after chemically induced inflammation compared to controls. However, in the same study, larvae immersed with several strains of *Lactobacillus plantarum* (WCFS1 and NA7) or other *Lactobacillus fermentum* strains (ATCC9338 and NA6) did not show differences in the expression of the same genes [53]. However, our study obtained a different pattern of gene expression for LHPro, LHPost and LPPost, underlining that not only the strain but the culturing process of production for both probiotic and postbiotics may affect the impact of the microbial product on innate effector cytokine expression profiles. LHPro induced significantly more *tnf*α gene expression than its inactivated counterpart, while *il17a* gene expression was up-regulated with LPPost when compared to LHPost. Interestingly, we observed a significant up-regulation of *il22* and *infγ* gene expression with LPPost, and a trend for an increase in *il22* gene expression with LHPro and LHPost when compared to the control. 

The cytokines IL-22 and IFN-γ belong to the class II cytokine family and are associated with Th17- and Th1-type cell-mediated immune responses, respectively. IL-22 has emerged as a key regulator of mucosal homeostasis and mediator of host defense in the intestine and elsewhere. Indeed, IL-22 is produced by both innate and adaptive immune cells and specifically targets epithelial cells, providing a circular link between immunity and mucosal homeostasis. The impact of pro- and postbiotics on *il22* gene expression is consistent with the increase in villi length, GCC and GCD, as well as the higher production of antimicrobial peptides, including lysozyme, and the gene expression of *zo2a*. In humans, Qiu et al. [54] reported that *L. plantarum* was able to induce IL-22 gene and protein expression in NK cells and increase the ZO-1 and occludin gene and protein expressions of colon epithelial cells subjected to challenge. Other authors found that live but not heat-inactivated *L. reuteri* D8 induced higher IL-22 secretion leading to STAT3 activation, which resulted in increased intestinal cell proliferation and elevated numbers of Paneth cells both in vitro (organoids) and in vivo (rodent models) [55]. Interestingly, those authors showed that heat-inactivated *L. reuteri* D8, *L. acidophilus* ATCC 4356, several MAMPs and microbiota metabolites could not significantly stimulate the secretion of IL-22, demonstrating strain-specific responses and the importance of live vs. heat inactivation. 

The current study revealed that zebrafish intestine expressed higher gene expression of *il22* and AMPs with the addition of LHPro, LHPost and LPPost. Concomitantly, we saw an increase in the number of IELs, among which were innate lymphoid cells type 3 (ILC3), known to be an important IL-22 producer in mammals as well as in zebrafish, and to express conserved factors like *rorc* and *il23* [56]. Such findings strengthen the comparisons that can be made between these model fish species and mammals and suggest that the addition of pro- and postbiotics leads to different IL-22 associated responses. However, while the role of IL-22 in maintaining gut homeostasis is now well-established, and although our results evidenced an effect of postbiotics on *il22* gene expression concomitantly to the improvement of gut barrier function biomarkers, more is still required to prove a direct cause–effect relationship between the induction of higher *il22* gene expression and the effect on gut barrier immune fate. This said, the observed elevations in IELs would suggest that, in the absence of infection, the inclusion of probiotics and postbiotics has a positive effect on maintaining IELs levels that help sustain barrier function either via maintaining the expression of junctional molecules or by producing cytokines to help maintain barrier integrity.

IFN-γ gene expression was stimulated when fish received the LPPost. Similarly, Maeda et al. [57] and Arimori et al. [58] found that daily intake of another strain of inactivated *L. plantarum* (strain L-137) resulted in increased expression and concentration of type I interferon in rodents, humans and pigs. The authors also demonstrated that such an effect could confer a better immune response against an *influenza* infection. Ou et al. [59] reported that heat-inactivated *E. faecalis YM-73* and *L. salivarius AP-32* show the potential to enrich the cytokine milieu with Th1-associated cytokines (IFN-γ) while reducing Th2-associated cytokines (IL-4), using an in vitro model with Caco2-cells. This study measured the gene expression of *ifnγ-1*, which has been found to be associated with ILC1-like cells rather than with NK cells in zebrafish [56], and is thus in agreement with the hypothesis that adding LPPost stimulates the activity of IELs, which encompass T-cell receptors γδ+ and ILC1 cells. However, we also observed a concomitant increase in the percentage of CD8^+^ T cells, especially with the LPPost diet, so the increase in *ifnγ* gene expression could be associated with this population of cells. 

The mechanisms by which pro- and postbiotic bacteria may act are complex and diverse, making the characterization of each strain important to advance the concepts of personalized nutrition and immunonutrition. First, various surface components have immunomodulatory effects, likely through interaction with PRRs on epithelial and immune cells, including dendritic cells, macrophages and lymphocytes [60]. It is increasingly accepted that cell components derived from lactobacilli are critical for their immunomodulatory effects, and that these effects are not necessarily dependent on the bacteria being alive [61,62]. On the contrary, in the study of Hou et al. [55], the induction of IL-22 by *L. reuteri* D8 was attributed to the production of a tryptophan metabolite, the indole-3-aldehyde, which regulates barrier function and immunity via the Aryl hydrocarbon receptor (AhR). Another study demonstrated that secretions from *L. acidophilus* could preserve epithelial barrier function through the modulation of occludin and claudin-1 protein expression and reductions in paracellular permeability in IL-1β-induced intestinal epithelial cells [60]. Recently, extracellular vesicles (EVs) derived from probiotics have emerged as potential mediators of host immune response and anti-inflammatory effect. Intake of EV from *L. plantarum* Q7 resulted in the down-regulation of inflammatory cytokines and the improvement of bacterial diversity by regulating the TLR4-MyD88-NF-κB pathway in mice suffering from induced colitis [63]. Little is known about the composition of such EV in Gram-positive bacteria, and first studies tend to indicate that there is little consistency between the protein composition of the EVs from different *Lactobacillus* species [37]. Finally, a modulation of the gut microbiota cannot be excluded. As an example, in broiler chickens, Khonyoung et al. [64] reported that segmented filamentous bacteria were more numerous in all of the heat-inactivated *L. plantarum* 137 groups than the control. Those bacteria are well-described and seem to play an important role in maintaining gut homeostasis and stimulating the innate immune system. More research is required to make a link between each bacterial strain, implication of EVs and its effect on the host.

## 5. Conclusions

Taken together, our results indicate that dietary administration of pro- or postbiotics strengthens the gut barrier function and innate immunity of healthy zebrafish in a strain-specific and process-dependent way. With some differences in the response intensity, the three treatments led to increased intestinal villi length and proportion of IELs, reinforcement of the GC population and up-regulated expression of biomarkers of AMP production and tight junction zona-occludin 2a (*zo-2a*). In addition, LPPost had an impact on the adaptive immune response, and we hypothesized that it conferred the potential to drive Th17/ILC3 immunity, as suggested by its effect on the gene expression of *il22,* of different AMPs and expression of *zo2a*. Moreover, LPPost showed the potential to drive Th1/ILC1-like immunity, with a higher percentage of CD8^+^ cells and higher *ifnγ* gene expression. Lactobacilli that exert stimulatory activity in augmenting Th1 responses are expected to enhance protection against bacterial and viral infections as well as to have anticancer effects by enhancing cell-mediated immunity, thus paving the way for the development of targeted immunonutrition strategies for humans and animals. 

## Figures and Tables

**Table 1:** Formulation (g/kg) of the basal diet.

	Control Diet
Wheat meal	265.0
Fabameal	250.0
Soybean meal	150.0
SPC60	239.0
Rapeseed oil	60.0
Vitamin premix	10.0
Lysine HCL	9.0
DL methionine	7.0
Gelatin	10.0

**Table 2:** Proximate composition of the basal diet (control).

	Control	LPPro	LHPost	LPPost
Dry matter (DM; %)	90.1 ± 0.03	89.1 ± 0.09	89.9 ± 0.04	90.2 ± 0.12
Crude Protein (% DM)	39.6 ± 0.15	39.4 ± 0.23	39.9 ± 0.66	39.7 ± 0.73
Crude Lipid (% DM)	5.9 ± 0.09	5.3 ± 0.15	5.6 ± 0.05	5.9 ± 0.31
Ash (% DM)	4.0 ± 0.06	4.0 ± 0.05	4.1 ± 0.14	4.0 ± 0.01

**Table 3:** Primers used for transcriptional analysis showing gene name, functional annotation, accession number, primer sequence and efficiency.

Gene	Functional Annotation	Accession Number	Primer Sequence (5′-3′)	Efficiency
**Reference genes**				
*cops2*	COP9 signalosome subunit 2	NM_001002055.1	F-TCCAGATGTACACGCACAA RATCAGCCATCCCACATCCAC	2.0
*metap1*	methionyl aminopeptidase 1	NM_001025165.2	F-GACGAGGGAGCCAAGGATT R-TCTGTGAAGCCTGGTATCCG	2.0
**Mucosal barrier markers**				
*lyz*	lysozyme	NM_139180.1	F-ATGAAGGGCTGATGGATTGA R-GGTGGGTCTTAAACTGCTTTC	2.1
*cath l*	cathepsin L.1	NM_001002368.1	F-GGTGGACTGCTCTGGTTCTT R-CTCACCATCCTGGGCTTCAT	2.0
*zo-2a*	tight junction protein 2a (zona occludens 2)	NM_001201571.1	F-CCTTGCTACCCAGTCCAGAAG R-GACGCAGACCAACGCTTTC	2.0
*muc2.1*	mucin 2.1	XM_021470771	F-CTGTGCGGCTAAAGGATAATC R-CTCTGTGAGGCTGGGCAATC	2.1
**Effector cytokines**				
*tnfα*	tumor necrosis factor alpha	NM_212859.2	F-CCATAAGACCCAGGCAATCA R-GGCAGCCTGGAAGTGAAATTG	2.0
*il1β*	interleukin 1, beta	NM_212844.2	F-CGGAAGCAGCGACTTGAAAG R-AACACACAGGCTGAGCAGAA	2.1
*ifnγ (ifng)*	interferon gamma 1	NM_212864.1	F-CCCATCTTCCTGCGAATCCT R-GCTTCATCCACGCTGTCATTC	2.1
*il22*	interleukin 22	NM_001020792.1	F-TGGAATCAGACGAGCACACA R-GGCTGGAGTAGTCGTGTTTACA	2.1
*il17a*	interleukin 17a/f1	NM_001020787.1	F-ACATAACGAGAGCCTGTATCCT R-CCTCAACGCCGTCTATCAGA	2.0
*tgfβ*	transforming growth factor, beta 2	NM_194386.2	F-AGGACAACACTGAGACTGAGTA R-GCAGTAGGGCAGGTCATTGT	2.1

**Table 4:** Histomorphometry and goblet cell chemotyping of zebrafish fed in different experiment groups (n = 9/group; means ± standard deviation). Different superscript letters denote significant differences between experimental groups for each parameter (*p* < 0.05).

	Control	LHPro	LHPost	LPPost	*p*-Value
*Morphological parameter*					
Muscularis thickness (µm)	8.6 ± 1.9	9.9 ± 2.9	8.4 ± 1.7	10.2 ± 2.7	0.29
Laminar propria width (µm)	10.3 ± 1.6 ^a^	10.1 ± 0.8 ^ab^	8.5 ± 1.4 ^b^	9.0 ± 1.0 ^ab^	0.03
Villi length (µm)	114.7 ± 31.0 ^a^	158.6 ± 26.0 ^b^	137.2 ± 27.0 ^ab^	171.9 ± 47.0 ^b^	0.01
IEL density (n/100 Enterocytes)	9.0 ± 0.7 ^a^	11.7 ± 1.4 ^b^	15.9 ± 2.9 ^c^	11.7 ± 1.1 ^b^	0.01
Goblet cell density (n/200 µm)	12.0 ± 4.1 ^a^	11.3 ± 1.9 ^ac^	15.6 ± 4.4 ^bc^	13.7 ± 1.8 ^b^	0.02
Goblet cell coverage (%)	10.3 ± 2.2 ^a^	7.7 ± 2.4 ^b^	12.4 ± 7.3 ^abc^	17.1 ± 6.1 ^c^	<0.05
*Goblet cell chemotyping*					
Acido mucins (%)	66.0 ± 4.1 ^a^	70.3 ± 2.5 ^ab^	76.1 ± 3.1 ^bc^	79.3 ± 1.6 ^c^	<0.05
Neutral mucins (%)	17.9 ± 2.0 ^a^	15.0 ± 1.5 ^ab^	11.6 ± 2.0 ^b^	6.2 ± 1.0 ^c^	<0.05
Both acidic and neutral mucins (%)	16.1 ± 2.3	14.7 ± 1.2	12.2 ± 1.9	14.4 ± 1.2	0.50

## Data Availability

Restrictions apply to the availability of these data. Data were obtained from Lallemand and are available from Mark Rawling or Marion Schiavone with the permission of Lallemand Animal Nutrition.

## Supplementary Materials

The following supporting information can be downloaded at: https://www.mdpi.com/article/10.3390/microorganisms11122900/s1, Figure S1—Schematic showing the posterior intestine of zebrafish; Figure S2—Gating strategy used to determine specific populations of CD4^+^ and CD8α^+^ cells from a heterogenous population of cells extracted from the posterior intestine of zebrafish; Figure S3—Extracellular membrane vesicles are present in heat-inactivated Lactobacilli; Figure S4—Representative micrographs of posterior intestinal sections from each group; Figure S5—Correlation matrix plots showing the highest variable contributions to each dimension; Figure S6—Gene expression and protein marker for barrier protection and regulation.

## References

[1] Wells J.M., Brummer R.J., Derrien M., MacDonald T.T., Troost F., Cani P.D., Theodorou V., Dekker J., Méheust A., De Vos W.M. (2017). Homeostasis of the gut barrier and potential biomarkers. Am. J. Physiol. Liver Physiol..

[2] Schoultz I., Keita Å.V. (2019). Cellular and molecular therapeutic targets in inflammatory bowel disease—Focusing on intestinal barrier function. Cells.

[3] Riedel S., Pheiffer C., Johnson R., Louw J., Muller C.J. (2022). Intestinal barrier function and immune homeostasis are missing links in obesity and type 2 diabetes development. Front. Endocrinol..

[4] Zhao W., Ho H.E., Bunyavanich S. (2019). The gut microbiome in food allergy. Ann. Allergy Asthma Immunol..

[5] O’Neill C.A., Monteleone G., McLaughlin J.T., Paus R. (2016). The gut-skin axis in health and disease: A paradigm with therapeutic implications. BioEssays.

[6] Hill C., Guarner F., Reid G., Gibson G.R., Merenstein D.J., Pot B., Morelli L., Canani R.B., Flint H.J., Salminen S. (2014). Expert consensus document: The International Scientific Association for Probiotics and Prebiotics consensus statement on the scope and appropriate use of the term probiotic. Nat. Rev. Gastroenterol. Hepatol..

[7] Salminen S., Collado M.C., Endo A., Hill C., Lebeer S., Quigley E.M., Sanders M.E., Shamir R., Swann J.R., Szajewska H. (2021). The International Scientific Association of Probiotics and Prebiotics (ISAPP) consensus statement on the definition and scope of postbiotics. Nat. Rev. Gastroenterol. Hepatol..

[8] Liu Q., Yu Z., Tian F., Zhao J., Zhang H., Zhai Q., Chen W. (2020). Surface components and metabolites of probiotics for regulation of intestinal epithelial barrier. Microb. Cell Factories.

[9] Lebeer S., Vanderleyden J., De Keersmaecker S.C. (2010). Host interactions of probiotic bacterial surface molecules: Comparison with commensals and pathogens. Nat. Rev. Microbiol..

[10] Burger-van Paassen N., Vincent A., Puiman P.J., van Der Sluis M., Bouma J., Boehm G., Van Goudoever J.B., Van Seuningen I., Renes I.B. (2009). The regulation of intestinal mucin MUC2 expression by short-chain fatty acids: Implications for epithelial protection. Biochem. J..

[11] Lopetuso L., Graziani C., Guarino A., Lamborghini A., Masi S., Stanghellini V. (2017). Gelatin tannate and tyndallized probiotics: A novel approach for treatment of diarrhea. Eur. Rev. Med. Pharmacol. Sci..

[12] Kataria J., Li N., Wynn J.L., Neu J. (2009). Probiotic microbes: Do they need to be alive to be beneficial?. Nutr. Rev..

[13] Núria P., Berlanga M., Miñana-Galbis D. (2019). Health Benefits of Heat-Killed (Tyndallized) Probiotics: An Overview. Int. J. Mol. Sci..

[14] Taverniti V., Guglielmetti S. (2012). Health-promoting properties of *Lactobacillus helveticus*. Front. Microbiol..

[15] Mujagic Z., De Vos P., Boekschoten M.V., Govers C., Pieters H.J.H., De Wit N.J., Bron P.A., Masclee A.A., Troost F.J. (2017). The effects of Lactobacillus plantarum on small intestinal barrier function and mucosal gene transcription; a randomized double-blind placebo controlled trial. Sci. Rep..

[16] Paveljšek D., Ivičak-Kocjan K., Treven P., Benčina M., Jerala R., Rogelj I. (2021). Distinctive probiotic features share common TLR2-dependent signalling in intestinal epithelial cells. Cell. Microbiol..

[17] Mohseni A.H., Casolaro V., Bermúdez-Humarán L.G., Keyvani H., Taghinezhad S.S. (2021). Modulation of the PI3K/Akt/mTOR signalling pathway by probiotics as a fruitful target for orchestrating the immune response. Gut Microbes.

[18] Zhong Y., Wang S., Di H., Deng Z., Liu J., Wang H. (2022). Gut health benefit and application of postbiotics in animal production. J. Anim. Sci. Biotechnol..

[19] Li Y., Li Y., Cao X., Jin X., Jin T. (2017). Pattern recognition receptors in zebrafish provide functional and evolutionary insight into innate immune signalling pathways. Cell. Mol. Immunol..

[20] Brugman S. (2016). The zebrafish as a model to study intestinal inflammation. Dev. Comp. Immunol..

[21] National Research Council (2012). Nutritional Requirements of Fish.

[22] Association of Official Analytical Chemists—AOAC (2007). Official Methods of Analysis.

[23] Caruana J.C., Dean S.N., Walper S.A. (2021). Isolation and characterization of membrane vesicles from *Lactobacillus* species. Bio-Protocol.

[24] Rawling M., Schiavone M., Apper E., Merrifield D.L., Castex M., Leclercq E., Foey A. (2023). Yeast cell wall extracts from Saccharomyces cerevisiae varying in structure and composition differentially shape the innate immunity and mucosal tissue responses of the intestine of zebrafish (*Danio rerio*). Front. Immunol..

[25] Leclercq E., Pontefract N., Rawling M., Valdenegro V., Aasum E., Andujar L.V., Migaud H., Castex M., Merrifield D. (2020). Dietary supplementation with a specific mannan-rich yeast parietal fraction enhances the gut and skin mucosal barriers of Atlantic salmon (*Salmo salar*) and reduces its susceptibility to sea lice (*Lepeophtheirus salmonis*). Aquaculture.

[26] Adams A., Thompson K. (1990). Development of an enzyme-linked immunosorbent assay (ELISA) for the detection of *Aeromonas salmonicida* in fish tissue. J. Aquat. Anim. Health.

[27] Bustin S.A., Benes V., Garson J.A., Hellemans J., Huggett J., Kubista M., Mueller R., Nolan T., Pfaffl M.W., Shipley G.L. (2009). The MIQE Guidelines: Minimum Information for Publication of Quantitative Real-Time PCR Experiments. Clin. Chem..

[28] Toda H., Shibasaki Y., Koike T., Ohtani M., Takizawa F., Ototake M., Moritomo T., Nakanishi T. (2009). Alloantigen-specific killing is mediated by CD8-positive T cells in fish. Dev. Comp. Immunol..

[29] Toda H., Saito Y., Koike T., Takizawa F., Araki K., Yabu T., Somamoto T., Suetake H., Suzuki Y., Ototake M. (2011). Conservation of characteristics and functions of CD4 positive lymphocytes in a teleost fish. Dev. Comp. Immunol..

[30] R Core Team (2022). R: A Language and Environment for Statistical Computing.

[31] Röhmel J. (1996). Precision intervals for estimates of the difference in success rates for binary random variables based on the permutation principle. Biom. J..

[32] Dohoo I.R., Ducrot C., Fourichon C., Donald A., Hurnik D. (1997). An overview of techniques for dealing with large numbers of independent variables in epidemiologic studies. Prev. Vet. Med..

[33] Lê S., Josse J., Husson F. (2008). FactoMineR: An R package for multivariate analysis. J. Stat. Softw..

[34] Waśko A., Polak-Berecka M., Kuzdraliński A., Skrzypek T. (2014). Variability of S-layer proteins in *Lactobacillus helveticus* strains. Anaerobe.

[35] Schär-Zammaretti P., Ubbink J. (2003). The cell wall of lactic acid bacteria: Surface constituents and macromolecular conformations. Biophys. J..

[36] Kurata A., Kiyohara S., Imai T., Yamasaki-Yashiki S., Zaima N., Moriyama T., Kishimoto N., Uegaki K. (2022). Characterization of extracellular vesicles from *Lactiplantibacillus plantarum*. Sci. Rep..

[37] Dean S.N., Leary D.H., Sullivan C.J., Oh E., Walper S.A. (2019). Isolation and characterization of Lactobacillus-derived membrane vesicles. Sci. Rep..

[38] Humam A.M., Loh T.C., Foo H.L., Samsudin A.A., Mustapha N.M., Zulkifli I., Izuddin W.I. (2019). Effects of feeding different postbiotics produced by *Lactobacillus plantarum* on growth performance, carcass yield, intestinal morphology, gut microbiota composition, immune status, and growth gene expression in broilers under heat stress. Animals.

[39] Matsuki T., Pédron T., Regnault B., Mulet C., Hara T., Sansonetti P.J. (2013). Epithelial cell proliferation arrest induced by lactate and acetate from *Lactobacillus casei* and *Bifidobacterium breve*. PLoS ONE.

[40] Khonyoung D., Yamauchi K.E. (2019). Improved growth performance due to hypertrophied intestinal absorptive epithelial cells by heat-killed Lactobacillus sakei HS-1 in broiler chickens. J. Anim Sci..

[41] Patnaude L., Mayo M., Mario R., Wu X., Knight H., Creamer K., Wilson S., Pivorunas V., Karman J., Phillips L. (2021). Mechanisms and regulation of IL-22-mediated intestinal epithelial homeostasis and repair. Life Sci..

[42] Ni Y., Zhang Y., Zheng L., Rong N., Yang Y., Gong P., Yang Y., Siwu X., Zhang C., Zhu L. (2023). Bifidobacterium and Lactobacillus improve inflammatory bowel disease in zebrafish of different ages by regulating the intestinal mucosal barrier and microbiota. Life Sci..

[43] Jariwala R., Mandal H., Bagchi T. (2017). Indigenous lactobacilli strains of food and human sources reverse enteropathogenic *E. coli* O26: H11-induced damage in intestinal epithelial cell lines: Effect on redistribution of tight junction proteins. Microbiology.

[44] Deplancke B., Gaskins H.R. (2001). Microbial modulation of innate defence: Goblet cells and the intestinal mucus layer. Am. J. Clin. Nutr..

[45] Xie S., Zhao S., Jiang L., Lu L., Yang Q., Yu Q. (2019). *Lactobacillus reuteri* stimulates intestinal epithelial proliferation and induces differentiation into goblet cells in young chickens. J. Agric. Food Chem..

[46] Elahi S.S.M., Mirnejad R., Kazempoor R., Sotoodehnejadnematalahi F. (2020). Study of the Histopathologic Effects of Probiotic *Lactobacillus acidophilus* in Exposure to E. coli O157: H7 in Zebrafish Intestine. Iran. Red Crescent Med. J..

[47] Lu F., Li Y., Wang X., Hu X., Liao X., Zhang Y. (2021). Early-life polyphenol intake promotes Akkermansia growth and increase of host goblet cells in association with the potential synergistic effect of Lactobacillus. Food Res. Int..

[48] Zhou X., Zhang K., Qi W., Zhou Y., Hong T., Xiong T., Xie M., Nie S. (2019). Exopolysaccharides from Lactobacillus plantarum NCU116 enhances colonic mucosal homeostasis by controlling epithelial cell differentiation and c-Jun/Muc2 signalling. J. Agric. Food Chem..

[49] Mwangi J., Hao X., Lai R., Zhang Z.Y. (2019). Antimicrobial peptides: New hope in the war against multidrug resistance. Zool. Res..

[50] Schlee M., Harder J., Köten B., Stange E.F., Wehkamp J., Fellermann K. (2008). Probiotic lactobacilli and VSL#3 induce enterocyte beta-defensin 2. Clin. Exp. Immunol..

[51] Möndel M., Schroeder B.O., Zimmermann K., Huber H., Nuding S., Beisner J., Fellermann K., Stange E.F., Wehkamp J. (2009). Probiotic *E. coli* treatment mediates antimicrobial human β-defensin synthesis and fecal excretion in humans. Mucosal Immunol..

[52] Becker H.M., Apladas A., Scharl M., Fried M., Rogler G. (2014). Probiotic *Escherichia coli* Nissle 1917 and commensal *E. coli* K12 diferentially affect the infammasome in intestinal epithelial cells. Digestion.

[53] Rajakumari D., Viswanath B., Rani A.U. (2021). Zebrafish: A novel model organism to assess probiotics influence on growth and development. Recent Developments in Applied Microbiology and Biochemistry.

[54] Qiu Y., Jiang Z., Hu S., Wang L., Ma X., Yang X. (2017). *Lactobacillus plantarum* enhanced IL-22 production in natural killer (NK) cells that protect the integrity of intestinal epithelial cell barrier damaged by enterotoxigenic *Escherichia coli*. Int. J. Mol. Sci..

[55] Hou Q., Ye L., Liu H., Huang L., Yang Q., Turner J.R., Yu Q. (2018). Lactobacillus accelerates ISCs regeneration to protect the integrity of intestinal mucosa through activation of STAT3 signalling pathway induced by LPLs secretion of IL-22. Cell Death Differ..

[56] Hernández P.P., Strzelecka P.M., Athanasiadis E.I., Hall D., Robalo A.F., Collins C.M., Boudinot P., Levraud J.P., Cvejic A. (2018). Single-cell transcriptional analysis reveals ILC-like cells in zebrafish. Sci. Immunol..

[57] Maeda N., Nakamura R., Hirose Y., Murosaki S., Yamamoto Y., Kase T., Yoshikai Y. (2009). Oral administration of heat-killed *Lactobacillus plantarum* L-137 enhances protection against influenza virus infection by stimulation of type I interferon production in mice. Int. Immunopharmacol..

[58] Arimori Y., Nakamura R., Hirose Y., Murosaki S., Yamamoto Y., Shidara O., Ichikawa H., Yoshikai Y. (2012). Daily intake of heat-killed *Lactobacillus plantarum* L-137 enhances type I interferon production in healthy humans and pigs. Immunopharmacol. Immunotoxicol..

[59] Ou C.C., Lin S.L., Tsai J.J., Lin M.Y. (2011). Heat-killed lactic acid bacteria enhance immunomodulatory potential by skewing the immune response toward Th1 polarization. J. Food Sci..

[60] Plaza-Diaz J., Ruiz-Ojeda F.J., Gil-Campos M., Gil A. (2019). Mechanisms of action of probiotics. Adv. Nutr..

[61] Guo S., Gillingham T., Guo Y., Meng D., Zhu W., Walker W.A., Ganguli K. (2017). Secretions of *Bifidobacterium infantis* and *Lactobacillus acidophilus* Protect Intestinal Epithelial Barrier Function. J. Pediatr. Gastroenterol. Nutr..

[62] Adams C.A. (2010). The probiotic paradox: Live and dead cells are biological response modifiers. Nutr. Res. Rev..

[63] Hao H., Zhang X., Tong L., Liu Q., Liang X., Bu Y., Gong P., Liu T., Zhang L., Xia Y. (2021). Effect of extracellular vesicles derived from *Lactobacillus plantarum* Q7 on gut microbiota and ulcerative colitis in mice. Front. Immunol..

[64] Khonyoung D., Yamauchi K.E. (2012). Effects of heat-killed *Lactobacillus plantarum* L-137 on morphology of intestinal villi and epithelial cells in broiler chickens. J. Appl. Anim. Res..

