# Supplementary material for: Modulation of Zebrafish (Danio rerio) Intestinal Mucosal Barrier Function Fed Different Postbiotics and a Probiotic from Lactobacilli
Source: Microorganisms. 2023 Nov 30;11(12):2900. doi: 10.3390/microorganisms11122900 (PMC10745996; doi:10.3390/microorganisms11122900)
Supplement: Supplementary file 1 [file microorganisms-11-02900-s001.zip › microorganisms-2683089-supplementary.pdf]

Supplementary figures

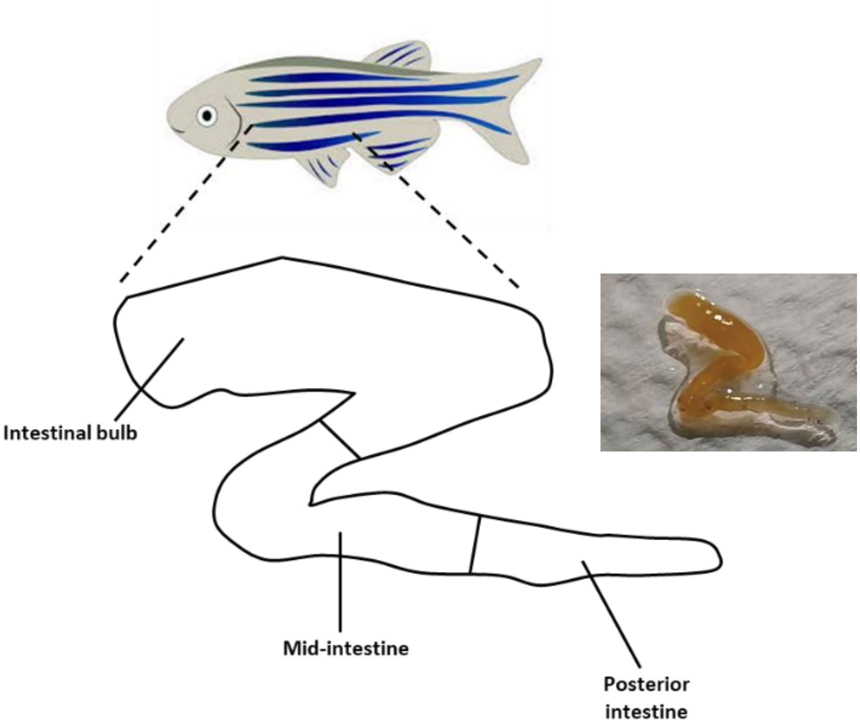

Figure S1. Schematic showing the posterior intestine of zebrafish.

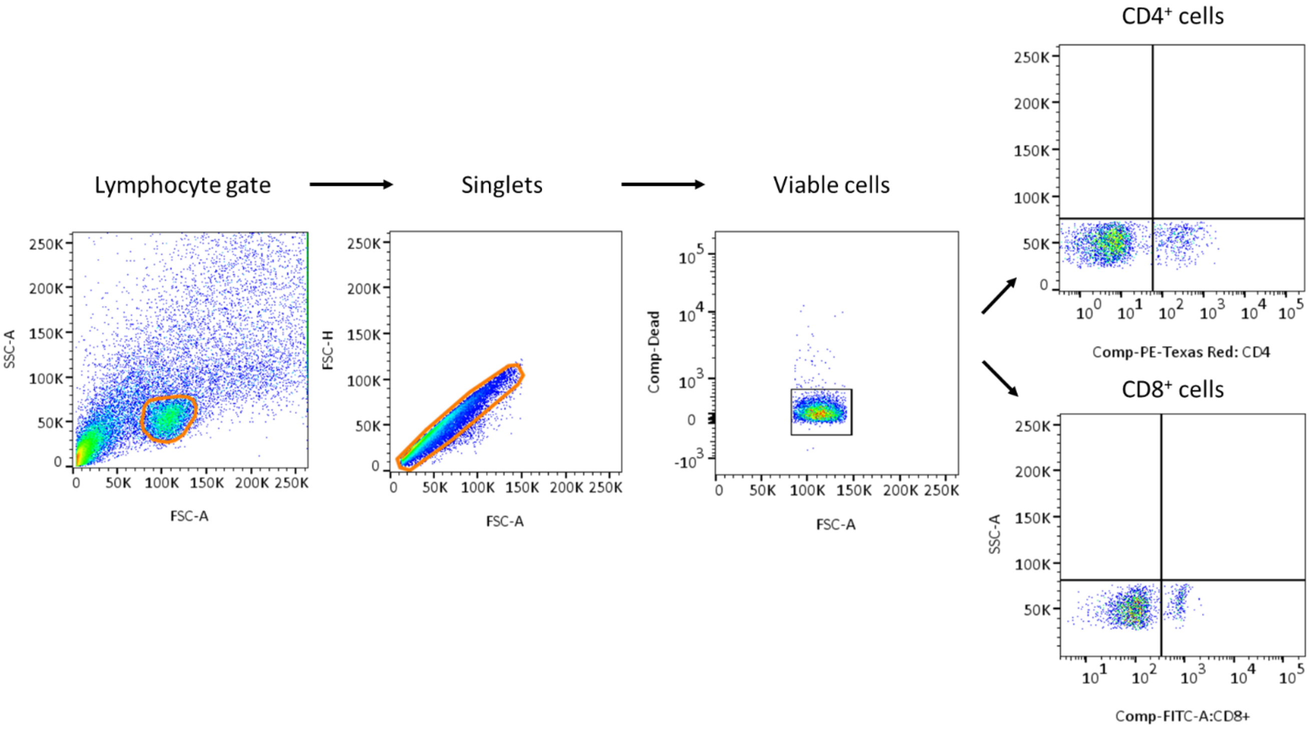

Figure S2. Gating strategy used to determine specific populations of $CD4^{+}$ and $CD8\alpha^{+}$ cells from a heterogenous population of cells extracted from the posterior intestine of zebrafish.

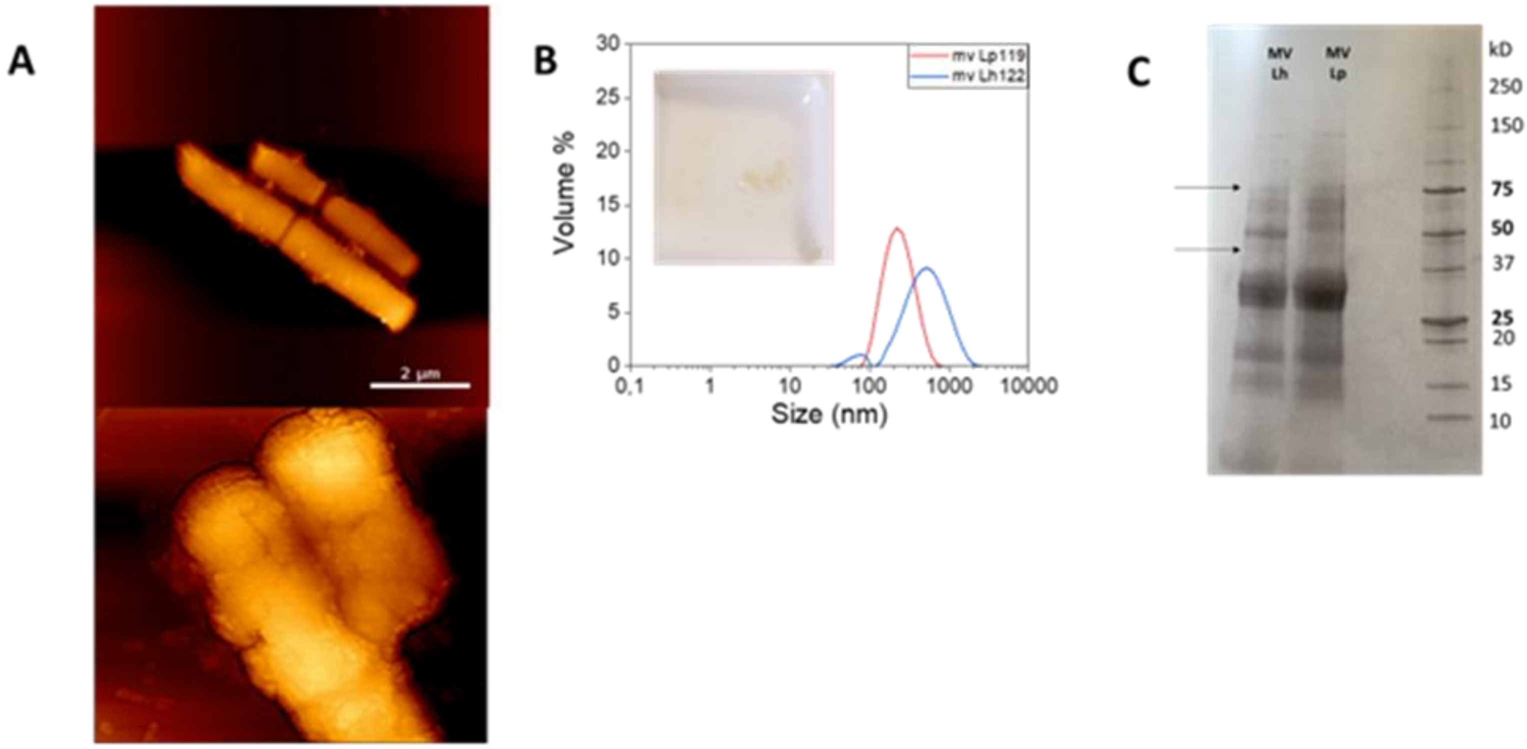

Figure S3. Extracellular membrane vesicles are present in heat-inactivated Lactobacilli. A) Atomic force microscopy (AFM) image of L. helveticus (Lh) HA-122 showing membrane vesicles (MVs). B) Size distribution of MVs assessed by DLS with a picture of MVs. C) Coomassie blue stained SDS-PAGE gel of purified MVs from MVs from L. helveticus (Lh) HA-122 and L. plantarum (Lp) HA-119. MVs with equal number of MVs were loaded.

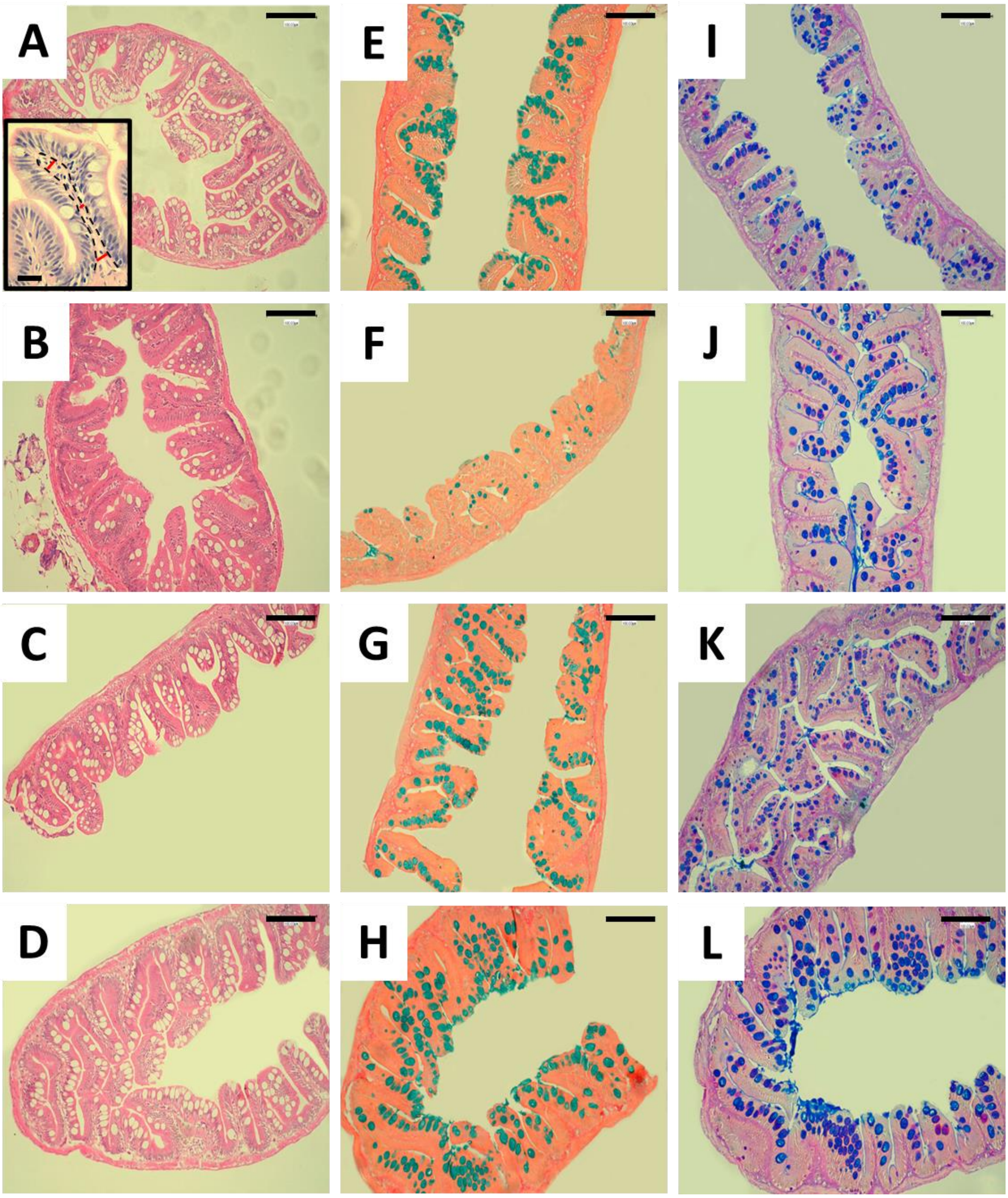

Figure S4. Representative micrographs of posterior intestinal sections from each group; A-I control group; B-J LHPro group; C-K LHPost group and D-L LPPost group. A-D display intestinal sections sained with Haematoxylin and Eosin and E-H display images stained with Alcian Blue van-Geison identifying goblet cells stained as blue colour. I-L display images stained with Ab-PAS identifying goblet cell chemotype, blue = acidic mucins, magenta = neutral mucins and purple = both acidic and neutral mucins. Sections were taken at 200 x magnification and the scale bar represents 100 µm. Image on top right in A, displays a micrograph of a representative villi indicating in red how the laminar propria width was calculated from an average of three measurements. Dashed lines display outline of laminar propria, and scale bar represents 10 µm at 400 x magnification.

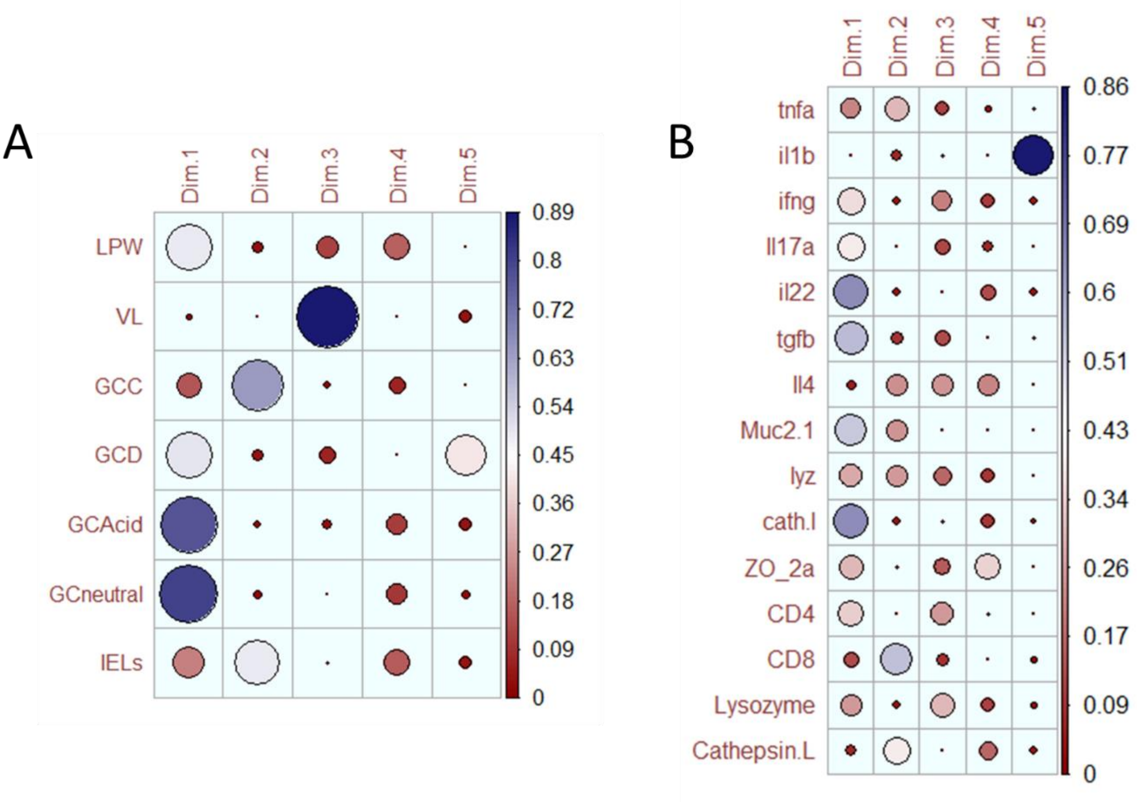

Figure S5. Correlation matrix plots showing the highest variable contributions to each dimension on A) PCA plot shown in Figure 2A; B) PCA plot shown in Figure 6A.

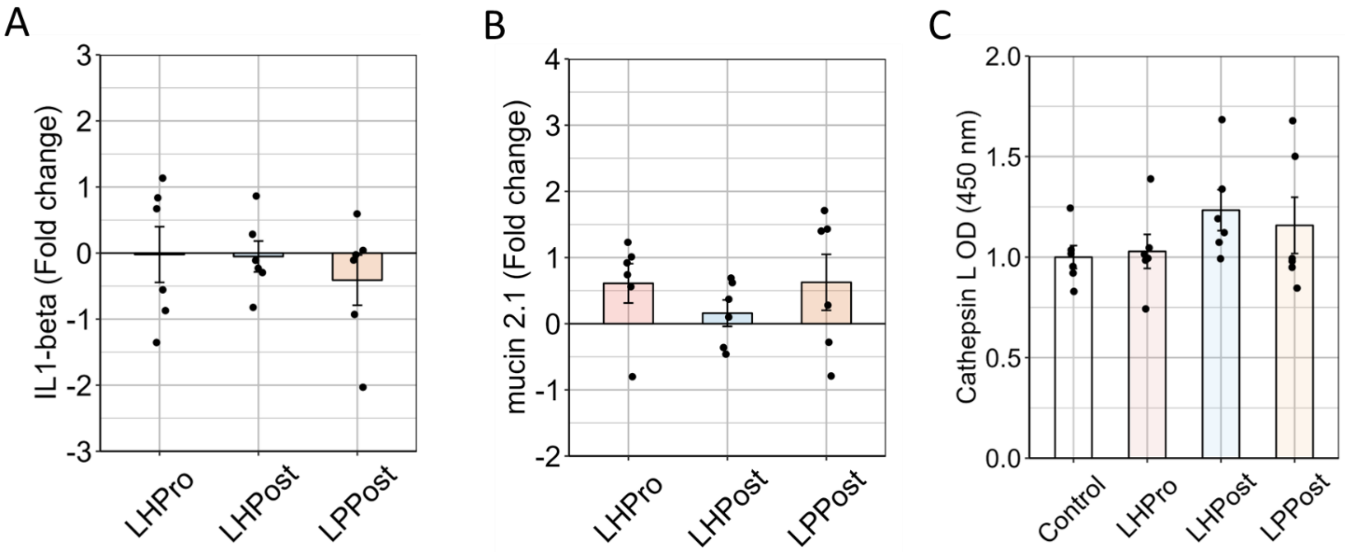

Figure S6. Gene expression and protein marker for barrier protection and regulation. A) *Il1β* gene expression (n=6/ treatment; log 2 fold change); B) *mucin2.1* gene expression (n=6/treatment; log 2 fold change; C) Cathepsin L protein expression as measured by ELISA (n=6/treatment).
